# Clonal transmission and species-specific mechanisms of polymyxin resistance in carbapenem-resistant *Enterobacteriaceae* from Southwest China

**DOI:** 10.3389/fcimb.2025.1678719

**Published:** 2025-10-13

**Authors:** Tingting Si, Hang Liu, Peiwen Xia, Na Huang, Shiyu Tang, Yuqiong Li, Qi Han, Yuanyuan Song, Yingyi Liu, Yun Xia

**Affiliations:** ^1^ Department of Laboratory Medicine, The First Affiliated Hospital of Chongqing Medical University, Chongqing, China; ^2^ Department of Clinical Laboratory, Institute of Translational Medicine, Renmin Hospital of Wuhan University, Wuhan, China

**Keywords:** carbapenem-resistant *Enterobacteriaceae*, polymyxin resistance, *mgrB* inactivation, *MCR-1*, fusion plasmid, clonal transmission

## Abstract

**Objectives:**

The rapid dissemination of carbapenem-resistant *Enterobacteriaceae* (CRE) poses a severe global health threat due to limited treatment options. Polymyxin is currently considered a last-resort therapy for human infections caused by CRE. The increasing clinical use of polymyxin has resulted in alarming resistance rates. This study investigated the clinical characteristics, molecular epidemiological characteristics, and resistance mechanisms of polymyxin-resistant CRE (PR-CRE) in Chongqing, China.

**Methods:**

Antimicrobial susceptibility and resistance genes were analyzed by PCR-based amplification and sequence analysis. Molecular epidemiological characteristics were analyzed using Enterobacterial Repetitive Intergenic Consensus (ERIC)-PCR and multilocus sequence typing (MLST). The gene expression of *pmrC* and *pmrK* was analyzed using qRT-PCR. Conjugation experiments were performed to determine plasmid transferability. Whole-genome sequencing (WGS) was performed to analyze their genetic environment.

**Results:**

Thirty PR-CRE isolates, including 21 *Klebsiella pneumoniae* and 9 *Escherichia coli*, exhibited multidrug resistance, with three pan-resistant *K. pneumoniae* strains. The predominant lineage was ST11-K64 *K. pneumoniae* (n=14/21), with four genetically identical isolates from ICUs, confirming clonal transmission. In contrast, all *E. coli* displayed high genetic diversity. Hypervirulence determinants were detected in 38.1% (n=8/21) of *K. pneumoniae*. Two rare *K. pneumoniae* strains were identified: one hypervirulent ST3984-KL64 strain co-harboring *bla*_KPC-2_ and *bla*_NDM-1_, and one ST2383-KL81 strain harboring *bla*_OXA-48_. Species-specific resistance mechanisms emerged: *K. pneumoniae* relied on chromosomal mutations in *mgrB*, *phoPQ*, and *pmrAB*, especially *mgrB* inactivation (57.1%, n=12/21) via IS*Kpn26*/IS*903B*/IS*Aeme19*/IS*Kpn14* and *pmrK* upregulation (95.2%, n=20/21), while *E. coli* exclusively used plasmid-borne *mcr-1* with 55.6% (n=5/9) conjugation efficiency, conferring low-level resistance. Genomic sequencing revealed that four identical IS*Aeme19* copies were first identified in ST11-KL64 hypervirulent CRKP-5: two on the chromosome (*mgrB* and *kdsD*), and two on plasmids (IncFII/IncR pkp2007-KPC and recombinant pkp2007-D). Transposition of IS*Aeme19* from pkp2007-KPC to *mgrB* was evidenced by the inverted orientation and matching flanking repeats. Crucially, a pan-resistant ST11-KL64 *K. pneumoniae* harbored a fusion plasmid with dual *bla*_KPC-2_ and *catA2* copies bracketed by IS*26*, a previously unreported configuration. Additionally, four novel deleterious mutations were detected: *mgrB*-Asn25Thr, *phoP*-Lys199Met, *phoQ*-Tyr89His, and *RamR*-Ala17Thr.

**Conclusion:**

These findings reveal species-divergent resistance mechanisms to polymyxin, necessitating enhanced surveillance of these high-risk clones, mobile elements, and emergent resistance mechanisms.

## 1 Introduction

In recent years, carbapenem-resistant *Enterobacteriaceae* (CRE) isolates, particularly carbapenem-resistant *Klebsiella pneumoniae* (CRKP) and *Escherichia coli* (CRECO), have emerged as formidable nosocomial pathogens (van Duin et al., 2020). Infections caused by CRE are associated with high mortality rates due to limited therapeutic options (Stewardson et al., 2019). Polymyxin is regarded as a last-resort agent for CRE infections; however, extensive clinical use has driven an alarming increase in resistance rates (Puljko et al., 2024; Xie et al., 2025). According to data from the China Antimicrobial Surveillance Network (CHINET, https://www.chinets.com/), the rate of polymyxin resistance among CRKP isolates has increased from 3.6% in 2020 to 11.8% in 2023. Furthermore, a systematic review revealed that the global prevalence of polymyxin-resistant *K. pneumoniae* was about 11.64% from 1987 to 2020, and the resistance rates reported in America, Europe, and Asia were 18.67%, 16.16%, and 10.17% (Sameni et al., 2022).

The primary mechanism of polymyxin resistance involves the modification of lipopolysaccharide (LPS) through the addition of cationic groups, specifically 4-amino-4-deoxy-L-arabinose (L-Ara4N) and/or phosphoethanolamine (PEtN). These modifications reduce the binding affinity of polymyxin and elevate the minimal inhibitory concentrations (MICs) (Binsker et al., 2021). This process is regulated by chromosomally encoded two-component regulatory systems (*phoPQ*, *pmrAB*, *and crrAB*) and the negative regulator *mgrB* (El-Sayed Ahmed et al., 2020). Additionally, plasmid-mediated mobile polymyxin resistance genes (*mcr-1* to *mcr-10*) induce outer membrane modifications by adding PEtN to lipid A (Rodríguez-Santiago et al., 2021; Liu et al., 2024). These genes demonstrate efficient horizontal transfer among humans, animals, and environments in more than 60 countries (Huang et al., 2020; Mmatli et al., 2022).

Furthermore, the convergence of polymyxin resistance and hypervirulent genes in CRKP isolates has fueled fatal hospital outbreaks globally, making molecular epidemiology essential for containment (Liu et al., 2022b). Given the complexity and diversity of these resistance mechanisms, our study aimed to investigate the molecular epidemiological characteristics and resistance mechanisms of polymyxin-resistant CRE (PR-CRE) isolates from Chongqing, China.

## 2 Materials and methods

### 2.1 Bacterial isolates and antimicrobial susceptibility testing

Clinical CRE isolates were collected from hospitalized patients at a tertiary teaching hospital in Chongqing, China. Species identification was initially performed using MALDI-TOF MS (BioMérieux, France). All isolates were stored at −80°C for subsequent analyses. MICs of polymyxin, tigecycline, and ceftazidime/avibactam were determined by broth microdilution according to the Clinical and Laboratory Standards Institute M100 (2024) guidelines. Additional antibiotics were tested using agar dilution. Tigecycline breakpoints followed the Food and Drug Administration criteria.

### 2.2 Detection of resistance genes, virulence genes and capsular serotyping

Carbapenemase genes (*bla*_KPC_ *, bla*_NDM_, *bla*_OXA-48,_ *bla*_VIM_, and *bla*_IMP_), *β*-lactamase genes (*bla*_TEM_, *bla*_SHV_, and *bla*_CTX-M_), virulence-associated genes (*iucA*, *iroB*, *p-rmpA*, *p-rmpA2*, and *peg-344*) and capsular serotyping in *K. pneumoniae* were detected by PCR and Sanger sequencing. The primers used can be found in **Supplementary Table 1**.

### 2.3 Multilocus sequence typing and enterobacterial repetitive intergenic consensus-PCR fingerprinting

Molecular epidemiological characteristics were assessed using MLST and ERIC-PCR. Cluster analysis was performed at 90% similarity using BioNumerics v7.6.3. MLST employed standard schemes: seven housekeeping genes (*ropB, gapA, mdh, pgi, phoE, infB*, and *tonB*) for *K. pneumoniae* and seven (*gyrB*, *icd*, *adk*, *mdh*, *fumC*, *purA*, and *recA*) for *E. coli*. Sequence types were assigned using the Pasteur Institute (http://bigsdb.pasteur.fr/*Klebsiella*/*Klebsiella*.html) and PubMLST (Carattoli et al., 2014) databases. The primers used are provided in **Supplementary Table 2**.

### 2.4 Polymyxin resistance mechanism characterization

Multiplex PCR was performed to detect *mcr-1* to *mcr-10*. Meanwhile, compared with the reference genomes of *K. pneumoniae* MGH78578 (NC_009648.1) and *E. coli* K12 MG1655 (NC_000913.3), chromosomal regulators (*pmrAB*, *phoPQ*, *crrAB*, *mgrB*) were amplified and sequenced. The impact of mutations was predicted using PROVEAN v1.1.5, with scores ≤ −2.5 considered deleterious. Insertion sequences disrupting the *mgrB* gene were identified using ISfinder (https://www-is.biotoul.fr/). Quantitative real-time PCR (qRT-PCR) was used to measure the expression of *pmrC* and *pmrK*, normalized to *rpsL* using the 2^−ΔΔCT^ method. A polymyxin-susceptible *K. pneumoniae* strain ATCC BAA-1705 was used as reference for the gene expression analysis. The primers used are provided in **Supplementary Table 3**. Conjugation assays transferred *mcr-1* to rifampicin-resistant *E. coli* EC600 on Mueller-Hinton agar containing polymyxin (2 mg/L) and rifampicin (400 mg/L). Subsequent antimicrobial susceptibility testing and PCR amplification confirmed plasmid transfer to the recipient.

### 2.5 Whole-genome sequencing and bioinformatics analysis

Genomic DNA was extracted and sequenced using PacBio RS II SMRT technology (Pacific Biosciences, USA) and Illumina HiSeq X Ten (Illumina, USA). Hybrid assembly was performed using Unicycler v0.4.8 (Wick et al., 2017), followed by error correction based on Pilon v1.24 (Walker et al., 2014). Gene annotation was conducted using Glimmer v3.0 (Delcher et al., 2007) and Prokka v1.11 (Seemann, 2014). Resistance genes, plasmid replicon types, and MLST were identified using the CGE platform (https://cge.cbs.dtu.dk/services/). Virulence factors, mobile elements, and plasmid transfer regions were detected using VFDB (Liu et al., 2022a), ISfinder (Siguier et al., 2006), and OriTfinder (Li et al., 2018) databases. Comparative genomics analysis was performed by *BLA*STn, BRIG (v1.3.0, https://sourceforge.net/projects/brig/) , and Easyfig (v2.2.5, https://mjsull.github.io/Easyfig/).

### 2.6 Statistical analysis

Statistical analysis was performed using GraphPad Prism 8 (GraphPad Software, San Diego, CA). With *K. pneumoniae* ATCC BAA-1705 as the reference, the expression levels of *pmrC* and *pmrK* in polymyxin-resistant *K. pneumoniae* strains were analyzed using unpaired two-tailed Student’s t-test. Statistical significance thresholds were defined as follows: *P < 0.05; **P < 0.01; ***P < 0.001.

## 3 Results

### 3.1 Antimicrobial susceptibility and clinical characteristics of PR-CRE isolates

**Figure 1:**
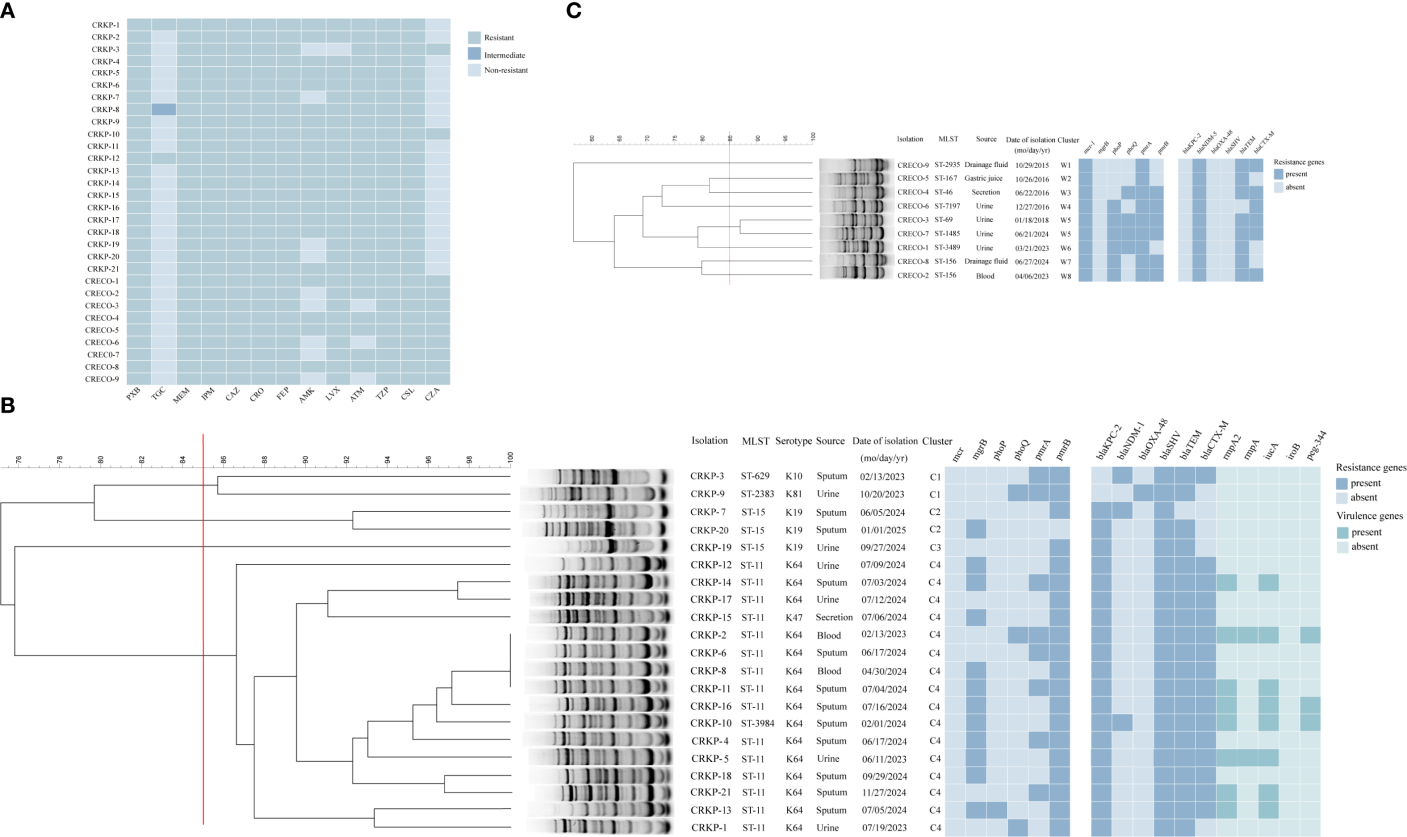
Characteristics of 30 polymyxin-resistant CRE isolates. **(A)** Heatmap showing polymyxin resistance profiles of 30 polymyxin-resistant CRE isolates (21 *Klebsiella pneumoniae*, 9 *Escherichia coli*); **(B)** ERIC-PCR dendrogram (≥85% similarity) of polymyxin-resistant CRKP strains. Strain isolations, multilocus sequence typing (MLST), setrotype, source of initial isolation, and data of isolation are included along each lane. The heatmap depict antimicrobial resistance genes and virulence genes of polymyxin-resistant CRKP isolates. **(C)** ERIC-PCR dendrogram (≥85% similarity) of polymyxin-resistant CRECO strains. Strain isolations, multilocus sequence typing (MLST) and data of isolation are included along each lane. The heatmap depict antimicrobial resistance genes of polymyxin-resistant CRECO isolates. The names of antimicrobial resistance genes are labeled at the top of this figure. Abbreviations: PXB, polymyxin B; TGC, tigecycline; MEM, meropenem; IPM, imipenem; CAZ,ceftazidime; CRO, ceftriaxone; FEP, cefepime; AMK, amikacin; LVX, levofloxacin; ATM, aztreonam; TZP, piperacillin-tazobactam; CSL, cefoperazone-sulbactam; CZA, ceftazidime-avibactam. Numbers in boldface type indicate susceptibility according to CLSI/EUCAST breakpoints.

All 30 polymyxin-resistant CRE isolates, including 21 polymyxin-resistant CRKP (PR-CRKP) and 9 polymyxin-resistant CRECO (PR-CRECO), exhibited multidrug resistance (**Figure 1A**). Polymyxin MICs for PR-CRKP isolates ranged from 4 to 128 µg/mL (MIC_50_ = 16 µg/mL, MIC_90_ = 64 µg/mL), with 71.4% (n=15/21) of the isolates exhibiting high-level resistance (MIC≥16 µg/mL). Three isolates (CRKP-1/8/12) exhibited co-resistance to tigecycline and polymyxin (**Supplementary Table 4**). Notably, most PR-CRKP isolates remained susceptible to ceftazidime/avibactam (90.5%, n=19/21) and tigecycline (85.7%, n=18/21). All PR-CRECO isolates displayed low-level polymyxin resistance (MIC range: 4–8 µg/mL) and were fully susceptible to tigecycline (**Supplementary Table 4**).

Patients infected with PR-CRKP were predominantly elderly males in ICUs (mean age: 66.7 years; 81.0% male). Sputum (57.1%, n=12/21) and urine (28.6%, n=6/21) were the primary specimen sources. Prior polymyxin exposure occurred in 66.7% (n=14/21) of cases, and 28.6% (n=6/21) experienced treatment failure (**Table 1**). Patients infected with PR-CRECO had a mean age of 54.1 years, with 44.4% (n=4/9) originating from urology departments and urine specimens accounting for 44.4% (n=4/9) of isolates (**Table 1**).

**Table 1:** Clinical characteristics of 30 patients infected with polymyxin-resistant CRKP isolates (n=21) and CRECO isolates (n=9).

Characteristics no. (%)	
CRKP	
Demographics	
Male	17(81.0)
Female	4(19.0)
Mean age (Min-Max)	66.7(34-90)
Department distribution	
Intensive Care Unit	15(71.4)
geriatric department	2(9.5)
Department of Cardiovascular Medicine	1(4.8)
Hematology ward	1(4.8)
Rehabilitation department	1(4.8)
Department of Nursing	1(4.8)
Type	
Sputum	12(57.1)
Urine	6(28.6)
Blood	2(9.5)
Secretion	1(4.8)
Use of polymyxin	14(66.7)
Death rate	6(28.6)
Hospitalization days median (Min-Max)	111.3(9-526)
CRECO	
Demographics	
Male	5(55.6)
Female	4(44.4)
Mean age (Min-Max)	54.1(17-85)
Department distribution	
Urinary Surgery	4(44.4)
Rehabilitation department	1(11.1)
Gastrointestinal Surgery	1(11.1)
Orthopedics	1(11.1)
Gynecology	1(11.1)
Department of Digestion	1(11.1)
Type	
Urine	4(44.4)
Drainage fluid	2(22.2)
Blood	1(11.1)
Secretion	1(11.1)
Gastric juice	1(11.1)

### 3.2 Molecular characteristics of polymyxin-resistant CRE isolates

#### 3.2.1 Resistance determinants, virulence genes, and molecular epidemiological characteristics of *K. pneumoniae*

All 21 PR-CRKP isolates produced carbapenemases: *bla*_KPC-2_ (n=18), *bla*_NDM-1_ (n=1), *bla*_OXA-48_ (n=1), or co-harbored *bla*_KPC-2_ and *bla*_NDM-1_ (n=1). Furthermore, extended-spectrum *β*-lactamase (ESBL) genes *bla*_SHV_, *bla*_TEM_, and *bla*_CTX-M_ were found in 100% (n=21), 95.2% (n=20), and 76.2% (n=16) of the isolates. Hypervirulent biomarkers were identified in seven PR-CRKP isolates, with distributions as follows: *rmpA2* (n=8), *iucA* (n=8), *peg344* (n=3) and *rmpA* (n=2) (**Figure 1B**).

MLST identified five distinct sequence types (STs) among the 21 isolates: ST11 (n=15), ST15 (n=3), ST629 (n=1), ST2383(n=1), and ST3984 (n=1) (**Figure 1B**). ST2383 and ST3984 represent rare lineages. Genetic analysis indicated that ST3984 was a single-locus variant of ST11 in *rpoB*, suggesting that ST3984 may be derived from ST11. Capsular genotyping revealed five KL types: KL64 (n=15), KL19 (n=3), KL47 (n=1), KL10 (n=1), and KL81 (n=1) (**Figure 1B**). Fourteen of fifteen ST11 isolates harbored KL64, while one ST11 isolate harbored KL47. Interestingly, KL64 was also present in the ST3984 isolate (CRKP-10). All ST15 isolates possessed KL19, whereas ST2383 and ST629 corresponded to KL81 and KL10, respectively.

ERIC-PCR clustering analysis (**Figure 1B**) delineated four major clusters (C1-C4) at >85.0% similarity. Cluster C4 (15 ST11 and 1 ST3984) was predominantly ICU-associated, which has emerged as the dominant and widely disseminated lineage regionally, with most isolates sharing >90.0% similarity. Within C4, four ICU isolates (CRKP-2/6/8/11) exhibited 100.0% genetic identity, confirming clonal transmission.

#### 3.2.2 Antimicrobial resistance determinants and molecular epidemiological characteristics of *E. coli*

PCR analysis confirmed that all PR-CRECO isolates produced *bla*_NDM-5_ carbapenemase (**Figure 1C**). Among *β*-lactamase genes, *bla*_TEM_ (n=8) and *bla*_CTX-M_ (n=6) were prevalent, while *bla*_SHV_ was undetected. The nine PR-CRECO isolates belonged to eight distinct STs and formed eight ERIC-PCR groups, with a similarity threshold of >85.0%, indicating high genetic diversity (**Figure 1C**).

### 3.3 Species-divergent resistance mechanisms to polymyxin

#### 3.3.1 Chromosome-mediated polymyxin resistance in *K. pneumoniae*

Given the absence of *mcr* genes among all PR-CRKP isolates, we focused on investigating chromosomal resistance mechanisms. Sequencing of key regulatory genes revealed missense mutations at seven sites in *pmrB*, four in *pmrA*, three in *phoQ*, and one in *phoP* (**Table 2**). Among these mutations, PROVEAN analysis predicted three *pmrB* mutations (Asp313Asn, Asp150Tyr, and Thr157Pro), one *pmrA* mutation (G53S), one *phoP* mutation (Lys199Met), and one *phoQ* mutation (Tyr89His) as deleterious. Notably, the *phoP* Lys199Met mutation in ST11 CRKP-13 strain and the *phoQ* Tyr89His mutation in ST11 CRKP-2 strain are previously unreported.

**Table 2:** Chromosomal mutations and plasmid-mediated genes related to polymyxin resistance in CRKP isolates.

Strain	Mutations in polymyxin resistance related genes^a^					Mcr
	*Mgrb*	*Phop*	*Phoq*	Pmra	*Pmrb*	
CRKP-1	WT	WT	785_787delGCG	WT	Thr246Ala; **Arg256Gly**	-
CRKP-2	WT	WT	**Tyr89His—**▲	**Gly53Ser**	Thr246Ala; **Arg256Gly**	-
CRKP-3	WT	WT	WT	665_666insC	Thr246Ala; **Arg256Gly**	-
CRKP-4	Complete deletion	WT	WT	665_666insC	Thr246Ala; **Arg256Gly**	-
CRKP-5	IS*Aeme19* insertion	WT	WT	WT	Thr246Ala; **Arg256Gly**	-
CRKP-6	WT	WT	WT	**Gly53Ser**; Glu223Lys	Thr246Ala; **Arg256Gly**; **Asp313Asn**	-
CRKP-7	WT	WT	WT	WT	396_407del12bp	-
CRKP-8	IS*903B* insertion	WT	WT	WT	Thr246Ala; **Arg256Gly**	-
CRKP-9	WT	WT	713delA	Glu57Gly	Thr246Ala	-
CRKP-10	**Asn25Thr**—▲	WT	WT	WT	Thr246Ala; **Arg256Gly**	-
CRKP-11	IS*kpn26* insertion	WT	WT	**Gly53Ser**	723_734del12bp; Thr246Ala; **Arg256Gly**	-
CRKP-12	IS*903B* insertion	WT	WT	WT	Thr246Ala **Arg256Gly**	-
CRKP-13	IS*kpn26* insertion	**Lys199Met—**▲	WT	WT	**Asp150Tyr;** Thr246Ala; **Arg256Gly**	-
CRKP-14	IS*kpn26* insertion	WT	WT	**Gly53Ser**	723_734del12bp; Thr246Ala; **Arg256Gly**	-
CRKP-15	169-179del11bp	WT	WT	WT	Thr246Ala **Arg256Gly**	-
CRKP-16	IS*kpn26* insertion	WT	WT	WT	Thr246Ala **Arg256Gly**	-
CRKP-17	WT	WT	WT	WT	Thr246Ala **Arg256Gly**	-
CRKP-18	IS*903B* insertion	WT	WT	WT	Thr246Ala **Arg256Gly**	-
CRKP-19	WT	WT	WT	WT	**Thr157Pro**	-
CRKP-20	IS*Kpn14* insertion	WT	WT	WT	WT	-
CRKP-21	WT	WT	WT	**Gly53Ser**	Thr246Ala **Arg256Gly**	-

^a^Genetic determinants of resistance were detected by performing PCR with gene-specific primers. Mutations were identified by comparison with wild-type reference sequences [*Klebsiella pneumoniae* MGH78578 (GenBank accession no. CP000647) for *pmrAB*, *phoPQ*, *mgrB*]. The mutations predicted as deleterious by PROVEAN were in bold and the novel mutation was marked with ▲. del, deletion; bp, base pair; W, wild-type. −, absence of target gene.

**Figure 2:**
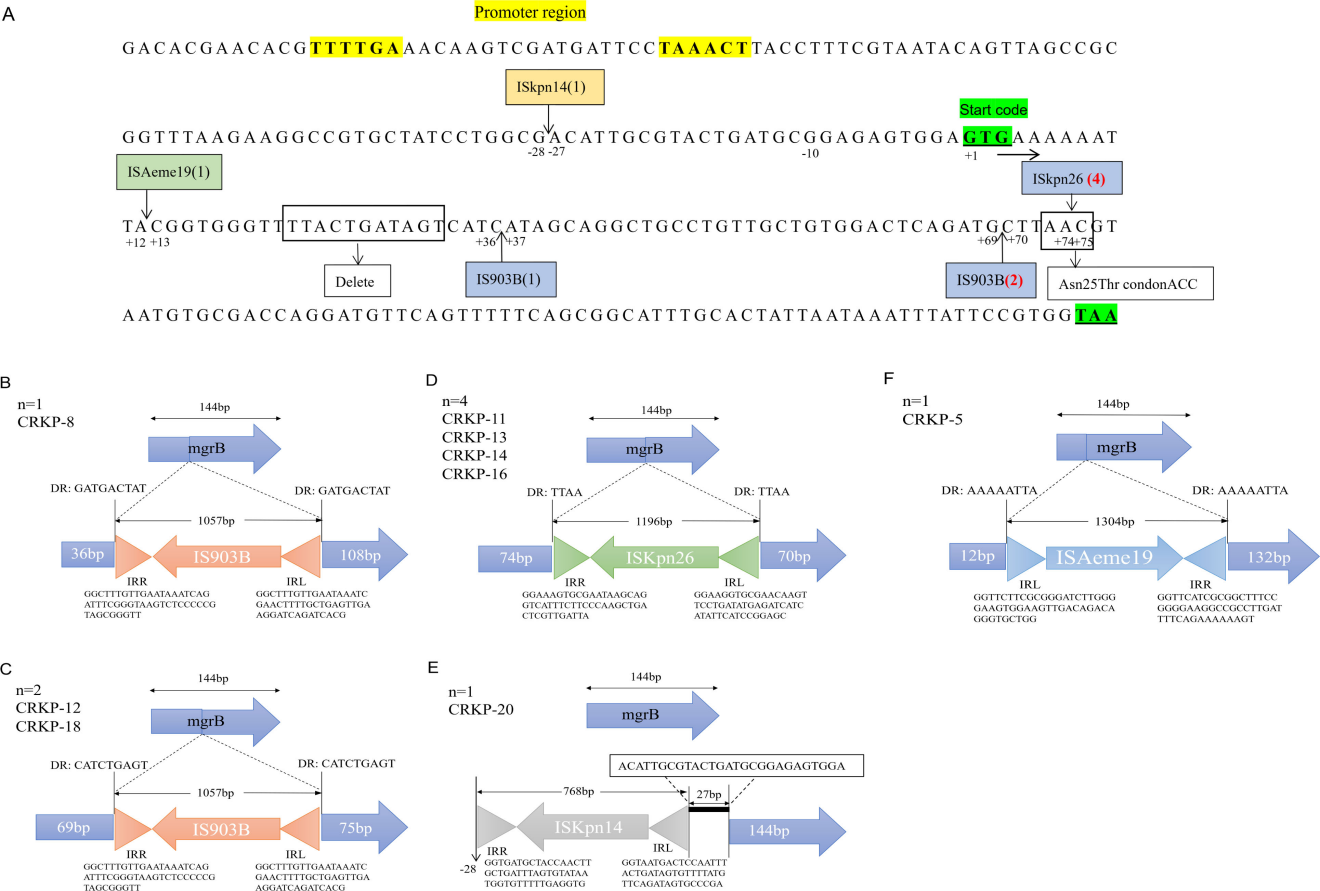
Alterations in the *mgrB* gene of polymyxin-resistant CRKP isolates. **(A)** Arrows indicate the positions of insertion mediate by IS elements. The number of insertions for each IS is given in parentheses. IS series are highlighted in different colors: blue, IS*5*; green, IS*L3*; orange, IS*1*. Boldsequences highlight the promoter region (without underline) and the start and stopcodons (both underlined). **(B)** One isolate with the IS*903B* insertion at position + 36. **(C)** Four isolates harboring the IS*Kpn26* insertion had insertions at position + 74. **(D)** Two isolates with the IS*903B* insertion at position + 69. **(E)** One had an insertion at -28 bp, located in the promoter region upstream of the start codon. **(F)** One isolate with the IS*Aeme19* insertion at position + 12. DR: direct repeat sequences; IRL/IRR: left/right inverted repeats (triangles).

Additionally, 57.1% (n=12/21) of isolates carried modified *mgrB*, including Asn25Thr mutation (n=1), complete or partial deletions (n=2), and insertional inactivation mediated by IS*Kpn26* (n=4), IS*903B* (n=3), IS*Aeme19* (n=1), and IS*Kpn14* (n=1) (**Table 2**; **Figure 2A**). Among the three isolates with IS*903B* (IS*5* family), one occurred at position  + 36 and two at +69 (**Figure 2B**; **Figure 2D**). IS*Kpn26* (IS*5* family) disrupted *mgrB* at nucleotide +74 in four isolates (**Figure 2C**). IS*Aeme19* (IS*L3* family) disrupted *mgrB* at nucleotide +12 in one isolate, while IS*Kpn14* (IS*1* family) caused upstream disruption (-28 nt) in one isolate (**Figure 2E**).

**Figure 3:**
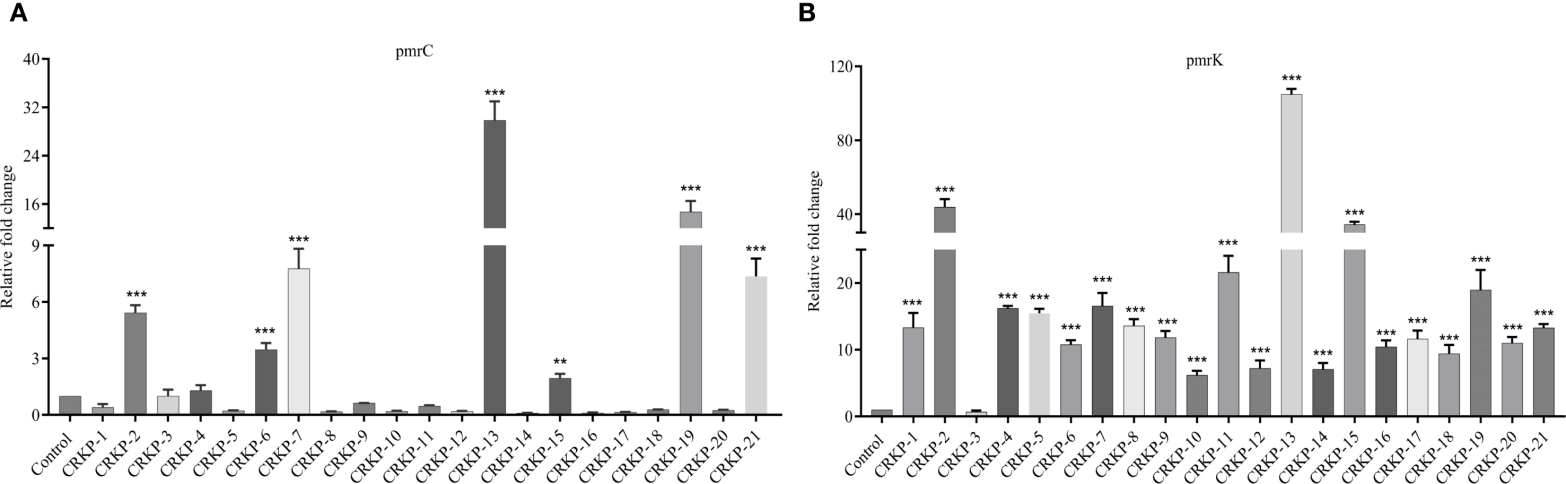
Expression analysis of LPS modification genes. Relative expressions of the pmrC **(A)** and pmrK **(B)** genes in polymyxin-resistant CRKP. Expression was normalized to *rpsL* using the susceptible *K. pneumoniae* ATCC BAA-1705 as control (expression = 1). (*P <0.05; **P < 0.01; ***P < 0.001).

To assess the functional impact of these genetic alterations, we measured the expression of *pmrC* and *pmrK* (components of the *pmrHFIJKLM* operon), which are key genes involved in LPS modification by adding PEtN and L-Ara4N. Compared with *K. pneumoniae* ATCC BAA-1705, 38.1% (n=8/21) of the isolates showed increased *pmrC* expression (1.29- to 29.86-fold) (**Figure 3A**), while 95.2% (n=20/21) exhibited upregulation of *pmrK* (5.89- to 104.85-fold) (**Figure 3B**).

#### 3.3.2 Plasmid-mediated resistance and conjugation assay of *mcr* in *E. coli*

All PR-CRECO isolates exhibiting low-level resistance harbored the *mcr-1* gene (**Figure 1C**). Notably, these isolates possessed the wild-type *mgrB* gene. PROVEAN analysis indicated that all amino acid substitutions in *pmrAB* and *phoPQ* were neutral (**Supplementary Table 5**). Conjugation assays confirmed the successful transfer of *mcr-1*-bearing IncI2 and IncHI2 plasmids from five isolates (CRECO-3, CRECO-4, CRECO-5, CRECO-6, and CRECO-7) to *E. coli* EC600, resulting in 4-fold to 8-fold increases in polymyxin MICs among transconjugants (**Table 3**). These findings suggest that IncHI2 and IncI2 plasmids are key vectors for the dissemination of *mcr-1* in PR-CRECO.

**Table 3:** Antimicrobial susceptibility profiles and plasmid replicon of recipient and transconjugants that received the polymyxin resistance gene *mcr-1*.

Isolates	MICs (µg/mL)^b^										Plasmid replicon		
	POL	TGC	MEM	IPM	CAZ	CRO	AMK	LVX	ATM	CZA	IncHI2	Inci2	Incx4
Recipient													
E. coli C600	0.5	0.125	0.0625	0.125	≤0.5	≤0.25	1	0.25	≤0.25	≤0.5/4	–	–	–
Transconjugants													
EC600/CRECO-3	**4**	0.125	0.0625	0.125	**16**	**128**	1	0.25	**64**	≤0.5/4	–	–	–
EC600/CRECO-4	**4**	0.125	0.0625	0.125	≤0.5	≤0.25	1	0.25	≤0.25	≤0.5/4	–	**+**	–
EC600/CRECO-5	**2**	0.125	0.0625	0.125	≤0.5	≤0.25	1	0.25	≤0.25	≤0.5/4	–	**+**	–
EC600/CRECO-6	**4**	0.125	0.0625	0.125	1	**32**	1	0.25	**4**	≤0.5/4	**+**	–	–
EC600/CRECO-7	**4**	0.125	0.0625	0.125	≤0.5	≤0.25	1	0.25	≤0.25	≤0.5/4	–	**+**	–

PXB, polymyxin B; TGC, tigecycline; MEM, meropenem; IPM, imipenem; CAZ, ceftazidime; CRO, ceftriaxone; AMK, amikacin; LVX, levofloxacin; ATM, aztreonam; CZA, ceftazidime-avibactam.

Numbers in boldface type indicate resistance according to CLSI/EUCAST breakpoints. +, presence of plasmid replicon.

### 3.4 Genomic features of IS*Aeme19*-mediated transposition and plasmid-chromosome exchange in CRKP-5

**Figure 4:**
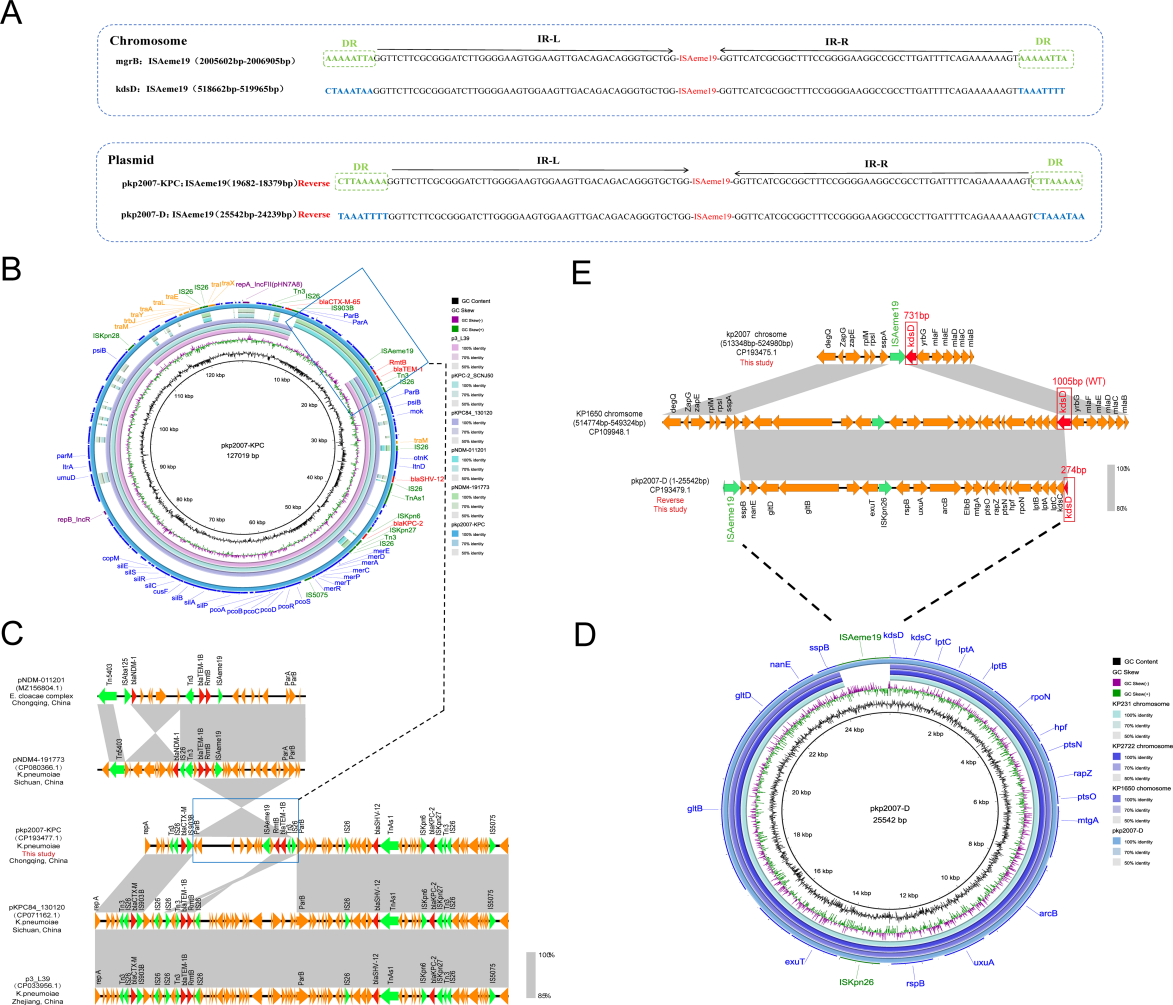
Genomic architecture and dynamics of *ISAeme19* in CRKP-5. **(A)** Structural organization of IS*Aeme19* elements. Each element contains 19-bp imperfect terminal inverted repeats (IR-L/R). Chromosomal copies (inserted in *mgrB* and *kdsD*) and plasmid copies (pkp2007-KPC, pkp2007-D) exhibit inverted orientations. Flanking sequences: 8-bp direct repeats (DRs) at *mgrB* and pkp2007-KPC sites vs. AT-rich regions at *kdsD* and pkp2007-D sites. Resistance genes (red), insertion sequences (green). **(B)** Circular map of plasmid pkp2007-KPC. BLASTn alignment reveals >99% identity with epidemic IncFII(pHN7A8)/IncR plasmids: pKPC84_130120 (Sichuan, China, CP071162.1), p3_L39 (Zhejiang, China, CP033956.1), and pKPC_SCNJ50 (Hunan, China, PP746481.1). Resistance genes (red arrows), insertion sequences (green arrows). **(C)** Homology analysis of the variable resistance region (IS*Aeme19*-*RmtB*-*bla*_TEM-1B_-Tn*3*-IS*26*) showing 99% coverage and 99.95% identity with *bla*_NDM_-harboring plasmids: pNDM-011201 (Chongqing, China, MZ156804.1) from the *Enterobacter cloacae complex* and pNDM4-19773 (Sichuan, China, CP080366.1) from ST16 *Klebsiella pneumonia*. Resistance genes are portrayed by red arrows. Green arrows indicate insertion sequences. **(D)** Formation mechanism of plasmid pkp2007-D via IS*Aeme19*-mediated excision of a 24-kb chromosomal segment. The excised region shares >99% identity with homologous segments in *K. pneumoniae* chromosomes KP1650/KP231/KP2722. **(E)** Sequence alignment analysis revealed that compared with KP1650 chromosome harboring the wild type *kdsD* gene (1005bp) between 514774bp and 549324bp, chromosome sequence of the kp2007 strain between 518657 bp and 518658 bp had a large fragment deletion and carried the truncated *kdsD* gene (731bp), which overlaps the gene fragments in pkp2007-D containing the deletion fragment (274bp) of the *kdsD* gene on the chromosome sequence of the kp2007. Mobile genetic elements (green), truncated/WT *kdsD* (red).

The CRKP-5 (ST11, KL64) isolate harbored a 5.49-Mb chromosome and six plasmids (5–195 kb), mainly including one virulence plasmid (pkp2007-VIR) and two multiple resistance plasmids (pkp2007-KPC and pkp2007-C) (**Supplementary Table 6**). The 195-kb IncHI1B(pNDM-MAR)/repB virulence plasmid pkp2007-VIR showed >99.9% identity to the prototypical pLVPK reference, carrying the aerobactin biosynthesis cluster (*iucABCD*-*iutA*) and mucoid phenotype regulators (*rmpA*, *rmpA2*) while lacking conjugation-related genes (**Supplementary Figure 1A**). The IncFII(pHN7A8)/IncR plasmid pkp2007-KPC closely resembled many published IncFII(pHN7A8)/IncR-type plasmids. Genetic environment analysis identified three multidrug resistance regions within this plasmid, which were composed of four IS*26* elements: (i) Tn*3*-IS*26*-*bla*_CTX-M-65_-IS*903B*; (ii) IS*Aeme19*-*rmtB*-*bla*_TEM-1B_-Tn*3*-IS*26*; (iii) IS*26*-*bla*_SHV-12_-Tn*As1*-IS*Kpn6*-*bla*_KPC-2_-IS*Kpn27*-Tn*3*-IS*26* (**Figure 4B**). The IncFII (pCRY) plasmid pkp2007-C backbone exhibited high conservation. It harbored two fused resistance regions (*dfrA14*-*sul2*-*tetA*-*catA2*-*qnrS1*-*bla*_LAP-2_) with complete modules characteristic of self-transmissible plasmids (**Supplementary Figure 1B**).

#### 3.4.1 Distribution and plasmid-chromosome transposition of IS*Aeme19*

Sequence comparison identified that CRKP-5 harbored four identical IS*Aeme19* copies: two on the chromosome (*mgrB* and *kdsD*), and two on plasmids (IncFII/IncR plasmid pkp2007-KPC and plasmid pkp2007-D), suggesting the active transposition capability of the IS*Aeme19* element (**Figure 4A**; **Table 4**). Crucially, the IS*Aeme19* element disrupting *mgrB* exhibited 100% sequence identity among the 1304 nucleotides, inverted orientation and identical 8-bp flanking direct repeats (DRs) to its counterpart on the multidrug resistance plasmid pkp2007-KPC, providing strong evidence for plasmid-to-chromosome transposition (**Figure 4A**). Evolutionary analysis of pkp2007-KPC further revealed inversion of a composite transposon (IS*Aeme19*-*rmtB*-*bla*_TEM-1B_-Tn*3*-IS*26*) relative to homologous *bla*_NDM_-harboring plasmids with inverted orientation and same 8-bp DRs on both sides of IS*Aeme19*, including pNDM-011201 (MZ156804.1, *Enterobacter cloacae complex*, Chongqing) and pNDM4-19773 (CP080366.1, *Klebsiella pneumoniae*, Sichuan) (**Figure 4B**; **Figure 4C**). These findings indicated that IS*Aeme19* may be inserted on pkp2007-KPC plasmid prior to its integration into the chromosome.

**Table 4:** Distribution and identity of four IS*Aeme19* copies in CRKP-5.

Subject acc.ver	% identity	Alignment length	Mismatches	Gap opens	Q. start	Q. end	S. start	S.end	Evalue	Bit score
Chromosome	99.693	1304	4	0	1	1304	518662	519965	0.0	2386
Chromosome	99.693	1304	4	0	1	1304	2005602	2006905	0.0	2386
pkp2007-KPC	99.693	1304	4	0	1	1304	19682	18379	0.0	2386
pkp2007-D	99.693	1304	4	0	1	1304	25542	24239	0.0	2386

#### 3.4.2 Chromosomal excision and plasmid formation mediated by IS*Aeme19*

The pkp2007-D was a 25,542 bp plasmid, containing 24 open reading frames, which did not contain any resistance genes and plasmid replicons. Genomic alignment confirmed pkp2007-D originated from the precise excision and recircularization of a 24-kb chromosomal segment (coordinates 518,657–542,201 bp) flanked by IS*Aeme19* elements, encompassing the truncated *kdsD* locus and adjacent genes (**Figure 4D**; **Figure 4E**), providing mechanistic evidence for IS*Aeme19*-driven excision and recircularization of chromosomal DNA into a stable episomal form lacking autonomous replication functions.

### 3.5 Genomic features of a fusion plasmid with dual *bla*_KPC-2_ copies and novel mutations in pan-resistant CRKP-1

To characterize polymyxin-tigecycline co-resistance, the pan-resistant CRKP-1 strain (ST11, KL64) was selected for WGS. A comprehensive summary of antimicrobial resistance genes and virulence-associated genes is presented in **Supplementary Table 7**.

**Figure 5:**
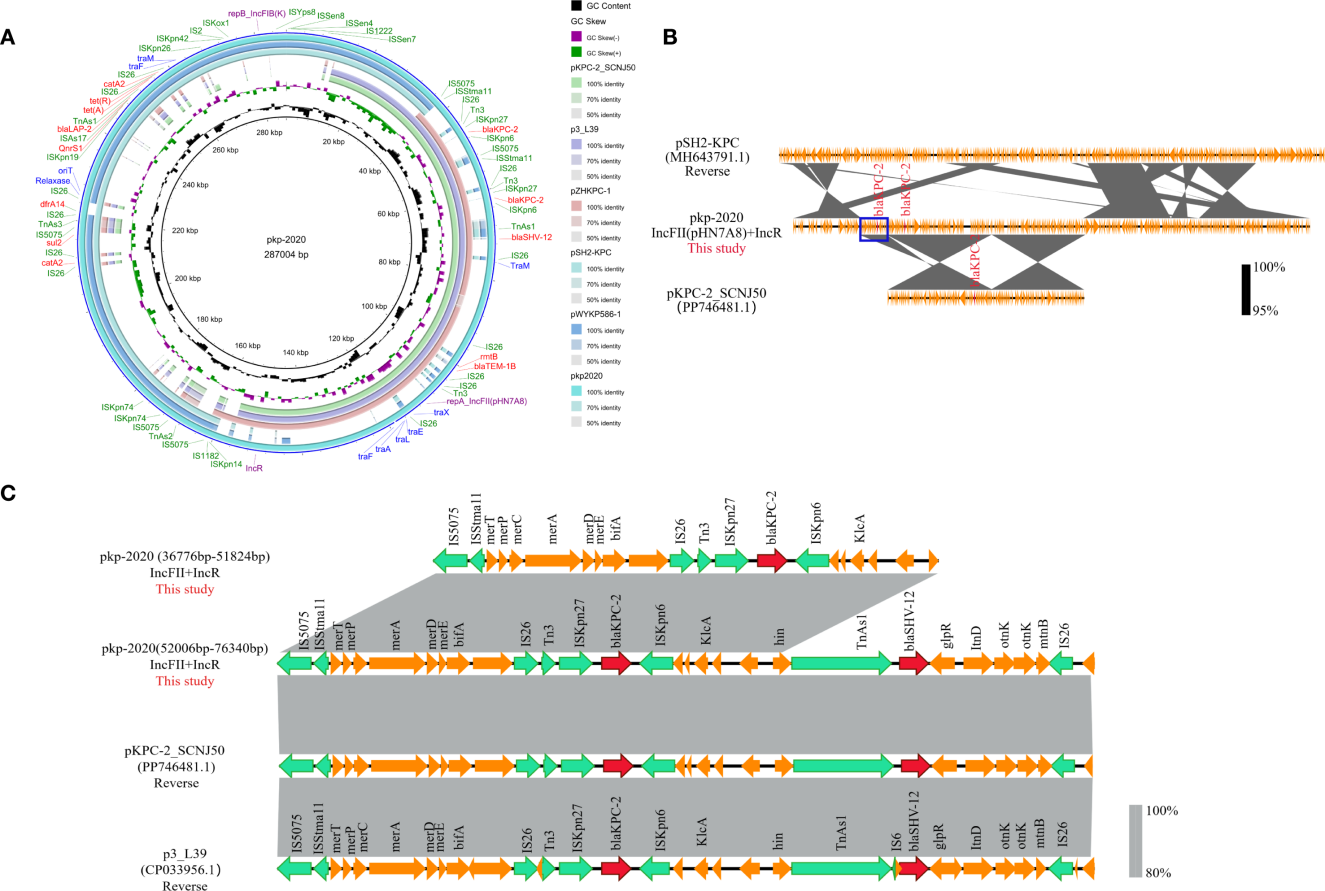
Genomic features of the fusion plasmid pKP2020 from CRKP-1. **(A)** Circular maps showing resistance genes (red), insertion sequences (green), replicons (purple), and conjugative modules (blue). **(B)** BLAST alignment of fusion plasmid pKP2020 harboring dual *bla*_KPC-2_ copies with related plasmids: pSH2-KPC and pKPC-2_SCNJ50. **(C)** Comparison of the regions harboring dual *bla*_KPC-2_ copies in pKP2020, pKPC-2_SCNJ50, and p3_L39. ORFs are colored: resistance (red), mobile elements (green), other accessory genes (yellow).

We identified that pan-resistant CRKP-1 strain harbored a large fusion plasmid pkp2020 (287,004 bp). *BLA*STn analysis revealed near-identical regions in pkp2020: the IncFII_pHN7A8_/IncR region shared 99.9–100.0% identity and 49.0–58.0% query coverage with plasmids pKPC-2_SCNJ50 (PP746481.1), p3_*L3*9 (CP033956.1), and pZHKPC-1 (OM928502.1), while the IncFIB_(K)_ segment exhibited 99.9–100.0% identity and 62.0% query coverage with plasmids pSH2-KPC (MH643791.1) and pWYKP586-1 (OQ801413.1) (**Figure 5A**). Sequence alignment indicated pkp2020 was a fusion product through recombination (**Figure 5B**). Crucially, pkp2020 contained an additional 15,048 bp multidrug resistance unit carrying *bla*_KPC-2_. Two direct-repeat *bla*_KPC-2_ copies resided 14,349 bp apart within the IncFII_pHN7A8_/IncR scaffold (**Figure 5C**).

In addition, this fusion plasmid carried 47 IS elements and 12 resistance genes, especially IS*26* (n=12/47), which mediates the insertion or recombination of resistance genes. Genetic environment analysis identified four multidrug resistance regions within this plasmid, composed of eleven IS*26* elements: (i) IS*26*-Tn*3*-IS*Kpn27*-*bla*_KPC-2_-IS*Kpn6*-ORF-IS*26*-Tn*3*-IS*Kpn27*-*bla*_KPC-2_-IS*Kpn6*-ORF-Tn*As1*-*bla*_SHV-12_-ORF-IS*26*; (ii) IS*26*-*rmtB*-*bla*_TEM-1B_-IS*26*; (iii) IS*26*-*catA2*-IS*26*-*sul2*-IS*5075*-Tn*As3*-IS*26*-*dfrA14*-IS*26*; (iv) IS*Kpn19*-*qnrS1*-IS*As17*-*bla*_LAP-2_-Tn*As1*-*tetA-tetR*-IS*26*-*catA2*-IS*26* (**Figure 5A**). Notably, pkp2020 lacked part of the *tra* gene region due to IS*26*-mediated truncation of plasmid conjugation genes during evolution.

Compared with the reference sequences from *E. coli* plasmid RP1 (X00006) for *tet(A)* and *K. pneumoniae* MGH78578 (CP000647) for other genes, CRKP-1 harbored *tet(A)* variants with Type 1 substitutions (I5R, V55M, I75V, T84A, S201A, F202S, V203F), IS*Kpn26*-mediated *acrR* inactivation, and unreported *RamR* mutations (Ala17Thr and *194Lys). PROVEAN predicted Ala17Thr as deleterious (**Supplementary Table 8**).

## 4 Discussion

Our findings reveal a striking divergence in polymyxin resistance trajectories: while *K. pneumoniae* evolved high-level resistance predominantly through chromosomal mutations (particularly IS*Kpn26*/IS*903B*/IS*Aeme19*/IS*Kpn14*-mediated *mgrB* inactivation) and the LPS modification gene (particularly *pmrK*) upregulation, *E. coli* evolved low-level resistance that relied exclusively on horizontally acquired *mcr-1*. Furthermore, the near-universal upregulation of *pmrK* (95.2% of isolates) versus *pmrC* (38.1% of isolates) in PR-CRKP isolates suggests that L-Ara4N addition is the dominant mechanism, with pEtN modification providing synergistic enhancement in a subset of CRKP strains. Previous studies have demonstrated that the Leu26Pro mutation in *PhoQ* (Cheng et al., 2015), Asp191Tyr mutation in *PhoP* (Jayol et al., 2015), and Thr157Pro mutation in *pmrB* (Jayol et al., 2014) were associated with polymyxin resistance. In our study, previously unreported deleterious substitutions (*mgrB*-Asn25Thr, *phoP*-Lys199Met, *phoQ*-Tyr89His, and *RamR*-Ala17Thr) expand the known mutational repertoire. These findings align with established evidence that chromosome-mediated mechanism is a primary cause of polymyxin resistance (Cannatelli et al., 2014; Zhu et al., 2023), and that polymyxin resistance likely occurs through increased expression of *pmrCAB* and *pmrHFIJKLM* operons, leading to lipid A modification (Nang et al., 2021).

This dichotomy underscores two distinct molecular epidemiological characteristics. First, the predominance of ST11-KL64 PR-CRKP aligns with its role as a major reservoir of carbapenem resistance in China (Liu et al., 2023), which was accelerated by IS element-mediated *mgrB* disruption (particularly IS*Kpn26*) under polymyxin selection pressure. Clonal transmission was confirmed by four genetically identical isolates from ICU patients, indicating persistent intra-hospital transmission despite infection controls. Second, plasmid-driven dissemination of *mcr-1* via IncHI2/IncI2 vectors occurred in genetically diverse *E. coli* isolates, with 55.6% conjugation efficiency underscoring the ongoing risk of horizontal transfer in community settings (Wang et al., 2017). This epidemiological bifurcation necessitates tailored infection control strategies: genomic surveillance should prioritize ST11-KL64 containment in ICUs while monitoring *mcr*-plasmid mobility.

Prior studies have associated virulence-resistance hybrids with elevated mortality in China (Hu et al., 2024b). In our study, two of six patients who experienced fatal outcomes were infected by ST11-KL64 strains that exhibited both high-level polymyxin resistance (MIC ≥ 16 μg/mL) and carried hypervirulence determinants. This suggests that the synergy between virulence and resistance contributes to unfavorable outcomes. Furthermore, two of three pan-resistant strains (CRKP-8, CRKP-12) were associated with treatment failure. These findings highlight an evolutionary trajectory toward untreatable infections, necessitating enhanced screening for virulence-resistance hybrids and pan-resistant strains in high-risk settings.

A recent study showed that the dissemination of IS*Kpn25* (IS*L3* family), which inserts into the *mgrB* gene at a specific site on pKpQIL plasmids, could explain the polymyxin resistance observed in clonally unrelated isolates harboring identical *mgrB* mutations (Giordano et al., 2018). IS*Aeme19* (IS*L3* family) is frequently located on plasmids carrying *bla*_NDM_ (Zhang et al., 2022; Sy et al., 2024). Although IS*Aeme19*-mediated *mgrB* inactivation has previously been reported only in ST16 *Klebsiella pneumoniae* from Southeast Asia (Sy et al., 2024; Nguyen et al., 2021), here we report the first case of IS*Aeme19*-mediated polymyxin resistance in an epidemic ST11-KL64 hypervirulent CRKP strain, involving a dual-targeting mechanism (disrupting both *mgrB* and *kdsD*) and a plasmid-chromosome recombination event. Critically, *kdsD* perturbation may compromise lipopolysaccharide core biosynthesis (Gunn and Ernst, 2007), potentially synergizing with *mgrB*-dysregulated lipid A modifications to establish a dual barrier against polymyxin. Given IS*Aeme19*’s transposition activity evidenced by identical 8-bp DRs flanking and inverted orientation of IS*Aeme19* between plasmid pkp2007-KPC and chromosomal *mgrB*, we propose that carbapenemase plasmids serve as Trojan horses for polymyxin resistance determinants in high-risk clones. This mechanism likely originated from *bla*_NDM_-bearing plasmids circulating in Southwest China (**Figure 4C**). Notably, pkp2007-KPC is an epidemic IncFII(pHN7A8)/IncR plasmid harboring *bla*_KPC-2_, indicating that IS*Aeme19* may co-disseminate with carbapenemase genes. Polymyxin therapy likely accelerates this process, as suggested by the emergence of resistance following treatment in our clinical case.

The fusion plasmid pKP2020 in pan-resistant ST11-KL64 CRKP-1 exemplifies alarming resistance consolidation. This fusion plasmid harbored duplicated *bla*_KPC-2_ and *catA2* copies bracketed by IS*26*. This unreported structure may amplify resistance through gene dosage effects during *β*-lactam exposure, potentially compromising ceftazidime/avibactam efficacy (Hu et al., 2024a). Concurrently, IS*26* recombinogenic hotspots facilitate modular acquisition of resistance genes (e.g., *catA2*, *rmtB*, and *qnrS1*), representing a critical evolutionary pathway for last-line antibiotic failure (He et al., 2015; Feng et al., 2019). Moreover, chromosomal mutations in CRKP-1, including the novel deleterious *RamR*-Ala17Thr substitution that likely dysregulates efflux pumps, synergized with plasmid-borne resistance, culminating in pan-drug resistance.

This study has several limitations. First, the small sample size of bacterial isolates necessitates expanded regional surveillance to validate the epidemiological patterns. Second, although the observed chromosomal mutations were associated with polymyxin resistance, their mechanistic roles require further functional validation.

## 5 Conclusions

This study reveals species-divergent resistance mechanisms to polymyxin. The persistence of *mcr-1* plasmids in diverse *E. coli* backgrounds highlights the ongoing risk of horizontal resistance spread. Meanwhile, the dominance of ST11-KL64 *K. pneumoniae* reliant on chromosomal adaptations and the emergence of novel genetic mechanisms (IS*Aeme19* transposition, fusion plasmids) underscore the continuous evolution of high-risk clones. While ceftazidime/avibactam retained activity against most PR-CRKP isolates, its vulnerability to porin mutations and *β*-lactamase amplification (e.g., dual *bla*_KPC-2_ copies in pkp2020) necessitates ongoing vigilance. Future studies should screen for IS*Aeme19*-like elements in high-risk plasmids and strengthen surveillance for these high-risk clones, mobile elements, and emergent resistance mechanisms to curb the spread of polymyxin-resistant CRE.

## Data Availability

The datasets presented in this study can be found in online repositories. The names of the repository/repositories and accession number(s) can be found in the article/**Supplementary Material**. The complete genome sequences of *K. pneumoniae* strains CRKP-1 and CRKP-5 have been deposited in the NCBI database under BioProject accession numbers PRJNA1286129 and PRJNA1268731.

## Supplementary material

Supplementary Figure 1 Comparative analysis of pkp2007-VIR and pkp2007-C with other similar plasmids in CRKP-5. Circular maps and genomic analysis of all insertion sequences distribution and antibiotic resistance genes. **(A)** BLASTn analysis showed that pkp2007-VIR backbone was similar to the classic virulence plasmid pLVPK,with over 99.94% nucleotide identity and 99% coverage ((e.g. CP103316.1, CP033955.1, CP054769.1 and CP146191.1). Virulence genes are indicated in red. Green arrows indicate insertion sequences. **(B)** BLASTn analysis showed that pkp2007-C was almost identical (100% query coverage, over 99.97% identity) to the plasmids pKPTCM2-3 (Zhejiang, China, CP118694.1), pKP12_4 (Zhejiang, China, CP082768.1), pHSKP43-3 (Shanghai, China, CP100101.1) and pHSKP8-3 (Shanghai, China, CP100090.1). ORFs encoding resistance genes are portrayed by red arrows. Green arrows indicate insertion sequences.
