# Supplementary material for: Clonal transmission and species-specific mechanisms of polymyxin resistance in carbapenem-resistant Enterobacteriaceae from Southwest China
Source: Front Cell Infect Microbiol. 2025 Oct 13;15:1678719. doi: 10.3389/fcimb.2025.1678719 (PMC12554700; doi:10.3389/fcimb.2025.1678719)
Supplement: Supplementary Figure 1 — Comparative analysis of pkp2007-VIR and pkp2007-C with other similar plasmids in CRKP-5. Circular maps and genomic analysis of all insertion sequences distribution and antibiotic resistance genes. (A) BLASTn analysis showed that pkp2007-VIR backbone was similar to the classic virulence plasmid pLVPK,with over 99.94% nucleotide identity and 99% coverage ((e.g. CP103316.1, CP033955.1, CP054769.1 and CP146191.1). Virulence genes are indicated in red. Green arrows indicate insertion sequences. (B) BLASTn analysis showed that pkp2007-C was almost identical (100% query coverage, over 99.97% identity) to the plasmids pKPTCM2-3 (Zhejiang, China, CP118694.1), pKP12_4 (Zhejiang, China, CP082768.1), pHSKP43-3 (Shanghai, China, CP100101.1) and pHSKP8-3 (Shanghai, China, CP100090.1). ORFs encoding resistance genes are portrayed by red arrows. Green arrows indicate insertion sequences. [file Table1.docx]

**Supplementary Table 1**. Primers for amplification of drug resistance gene，virulence genes and capsular serotyping.

|  | Primer name | Sequence (5′–3′) | Reference |
| --- | --- | --- | --- |
| Carbapenems | *bla*KPC-F | CGTCTAGTTCTGCTGTCTTG | (Poirel et al., 2011) |
|  | *bla*KPC-R | CTTGTCATCCTTGTTAGGCG |  |
|  | *bla*NDM-F | GGTTTGGCGATCTGGTTTTC |  |
|  | *bla*NDM-R | CGGAATGGCTCATCACGATC |  |
|  | *bla*IMP-F | GGAATAGAGTGGCTTAAYTC |  |
|  | *bla*IMP-R | GGTTTAAYAAAACAACCACC |  |
|  | *bla*VIM-F | GATGGTGTTTGGTCGCATA |  |
|  | *bla*VIM-R | CGAATGCGCAGCACCAG |  |
|  | *bla*OXA-48-F | GCGTGGTTAAGGATGAACAC |  |
|  | *bla*OXA-48 -R | CATCAAGTTCAACCCAACCG |  |
| β-lactamase genes | *bla*TEM-F | GTGCGCGGAACCCCTATT | (Quan et al., 2017) |
|  | *bla*TEM-R | TTACCAATGCTTAATCAGTGAGGC |  |
|  | *bla*SHV-F | CTTTACTCGCCTTTATCGGC |  |
|  | *Bla*SHV-R | TTACCGACCGGCATCTTTCC |  |
|  | *bla*CTX-M-F | GCTTTATGCGCAGACGAGTG |  |
|  | *bla*CTX-M-R | GCCAGATCACCGCAATATCA |  |
| Virulence genes detection | iucA-F | GCTTATTTCTCCCCAACCC | (Russo et al., 2018) |
|  | iucA-R | TCAGCCCTTTAGCGACAAG |  |
|  | iroB-F | ATCTCATCATCTACCCTCCGCTC |  |
|  | iroB-R | GGTTCGCCGTCGTTTTCAA |  |
|  | peg-344-F | CTTGAAACTATCCCTCCAGTC |  |
|  | peg-344-R | CCAGCGAAAGAATAACCCC |  |
|  | prmpA-F | GAGTAGTTAATAAATCAATAGCAAT |  |
|  | prmpA-R | CAGTAGGCATTGCAGCA |  |
|  | prmpA2-F | GTGCAATAAGGATGTTACATTA |  |
|  | prmpA2-R | GGATGCCCTCCTCCTG |  |
|  | peg-344-R | GCTTATTTCTCCCCAACCC |  |
|  | prmpA-F | TCAGCCCTTTAGCGACAAG |  |
|  | prmpA-R | ATCTCATCATCTACCCTCCGCTC |  |
| Capsular serotyping | wzi-F | CGAGCGCTTTCTATCTTGGT | This study |
|  | wzi-R | GAGAGCCACTGGTTCCAGAA |  |
| Plasmid typing | HI2 FW | TTTCTCCTGAGTCACCTGTTAACAC | (Carattoli et al., 2005) |
|  | HI2 RW | GGCTCACTACCGTTGTCATCCT |  |
|  | I2 FW | CTGTCGGCATGTCTGTCTC |  |
|  | I2 RW | CTGGCTACCAGTTGCTCTAA |  |
|  | X4 FW | AGCAAACAGGGAAAGGAGAAGACT | (Johnson et al., 2012) |
|  | X4 RW | TACCCCAAATCGTAACCTG |  |

**Supplementary Table 2**. Primers for amplification of Multilocus sequence typing (MLST) and ERIC-PCR fingerprinting.

| Primer name | Sequence (5′–3′) | Reference |
| --- | --- | --- |
| KP-rpoB-F | GGCGAAATGGCWGAGAACCA | (Diancourt et al., 2005) |
| KP-rpoB-R | GAGTCTTCGAAGTTGTAACC |  |
| KP-gapA-F | TGAAATATGACTCCACTCACGG |  |
| KP-gapA-R | CTTCAGAAGCGGCTTTGATGGCTT |  |
| KP-mdh-F | CCCAACTCGCTTCAGGTTCAG |  |
| KP-mdh-R | CCGTTTTTCCCCAGCAGCAG |  |
| KP-pgi-F | GAGAAAAACCTGCCTGTACTGCTGGC |  |
| KP-pgi-R | CGCGCCACGCTTTATAGCGGTTAAT |  |
| KP-phoE-F | ACCTACCGCAACACCGACTTCTTCGG |  |
| KP-phoE-R | TGATCAGAACTGGTAGGTGAT |  |
| KP-infB-F | CTCGCTGCTGGACTATATTCG |  |
| KP-infB-R | CGCTTTCAGCTCAAGAACTTC |  |
| KP-tonB-F | CTTTATACCTCGGTACATCAGGTT |  |
| KP-tonB-R | ATTCGCCGGCTGRGCRGAGAG |  |
| ECO-adk-F | TCATCATCTGCACTTTCCGC | (Tartof et al., 2005) |
| ECO-adk-R | CCAGATCAGCGCGAACTTCA |  |
| ECO-fumC-F | TCACAGGTCGCCAGCGCTTC |  |
| ECO-fumC-R | GTACGCAGCGAAAAAGATTC |  |
| ECO-gyrB-F | TCGGCGACACGGATGACGGC |  |
| ECO-gyrB-R | ATCAGGCCTTCACGCGCATC |  |
| ECO-icd-F | ATGGAAAGTAAAGTAGTTGTTCCGGCACA |  |
| ECO-icd-R | GGACGCAGCAGGATCTGTT |  |
| ECO-mdh-F | ATGAAAGTCGCAGTCCTCGGCGCTGCTGGCGG |  |
| ECO-mdh-R | TTAACGAACTCCTGCCCCAGAGCGATATCTTTC |  |
| ECO-purA-F | TCGGTAACGGTGTTGTGCTG |  |
| ECO-purA-R | CATACGGTAAGCCACGCAGA |  |
| ECO-recA-F | CGCATTCGCTTTACCCTGACC |  |
| ECO-recA-R | TCGTCGAAATCTACGGACCGGA |  |
| ERIC-F | ATGTAAGCTCCTGGGGATTCA | (Codjoe et al., 2019) |
| ERIC-R | AAGTAAGTGACTGGGGTGAGCG |  |

**Supplementary Table 3**. Primers for amplification of polymyxin resistance genes.

| Primer name | Sequence (5′–3′) | Reference |
| --- | --- | --- |
| KP-pmrA-F | CATTTCCGCGCACTGTCTGC | (Jayol et al., 2014) |
| KP-pmrA-R | CAGGTTTCAGTTGCAAACAG |  |
| KP-pmrB-F | ACCTACGCGAAAAGATTGGC |  |
| KP-pmrB-R | GATGAGGATAGCGCCCATGC |  |
| KP-phoP-F | GAGCTTCAGACTACTATCGA | (Cannatelli et al., 2013) |
| KP-phoP-R | GGGAAGATATGCCGCAACAG |  |
| KP-phoQ-F | ATACCCACAGGACGTCATCA |  |
| KP-phoQ-R | CAGGTGTCTGACAGGGATTA |  |
| KP-mgrB-F | AAGGCGTTCATTCTACCACC |  |
| KP-mgrB-R | TTAAGAAGGCCGTGCTATCC |  |
| KP-crrB-F | GGATTGAAGGGCATTCCGGA | (Pishnian et al., 2019) |
| KP-crrB-R | GCAGTATGTGGGATCTGTCT |  |
| ECO-mgrB-F | AAGGTAGGTGAAACGGAGATT | (Quan et al., 2017) |
| ECO-mgrB-R | CCGATACAACCAAAGACGC |  |
| ECO-phoP-F | ATGGCGATGCTGTCCG |  |
| ECO-phoP-R | TCCGTAGGCAAGCGAAA |  |
| ECO-phoQ-F | GCAAAGTGGTCAGCAAAGA |  |
| ECO-phoQ-R | AATCGGGCCAGTTAAGAGT |  |
| ECO-pmrA-F | TGCTGTGGCTGTCGGA |  |
| ECO-pmrA-R | AATCTGCTCGGTACTTTCATG |  |
| ECO-pmrB-F | CCAACACCCTGGAAGTGC |  |
| ECO-pmrB-R | TGATGAATAAGCTGAAACGGA |  |
| mcr-1-F | AGTCCGTTTGTTCTTGTGGC | (Rebelo et al., 2018) |
| mcr-1-R | AGATCCTTGGTCTCGGCTTG |  |
| mcr-2-F | CAAGTGTGTTGGTCGCAGTT |  |
| mcr-2-R | TCTAGCCCGACAAGCATACC |  |
| mcr-3-F | AAATAAAAATTGTTCCGCTTATG |  |
| mcr-3-R | AATGGAGATCCCCGTTTTT |  |
| mcr-4-F | TCACTTTCATCACTGCGTTG |  |
| mcr-4-R | TTGGTCCATGACTACCAATG |  |
| mcr-5-F | ATGCGGTTGTCTGCATTTATC |  |
| mcr-5-R | TCATTGTGGTTGTCCTTTTCTG |  |
| mcr-6-F | AGCTATGTCAATCCCGTGAT | (Borowiak et al., 2020) |
| mcr-6-R | ATTGGCTAGGTTGTCAATC |  |
| mcr-7-F | GCCCTTCTTTTCGTTGTT |  |
| mcr-7-R | GGTTGGTCTCTTTCTCGT |  |
| mcr-8-F | TCAACAATTCTACAAAGCGTG |  |
| mcr-8-R | AATGCTGCGCGAATGAAG |  |
| mcr-9-F | TTCCCTTTGTTCTGGTTG |  |
| mcr-9-R | GCAGGTAATAAGTCGGTC |  |
| mcr-10-F | CTCGCTTCGCTGATCCTGAT | (Portal et al., 2024) |
| mcr-10-R | CGCTGGTAATAGGTCGGTCC |  |
| pmrC-RT-F | GCGTGATGAATATCCTCACCA | (Wang et al., 2017) |
| pmrC-RT-R | CACGCCAAAGTTCCAGATGA |  |
| pmrK-RT-F | AGTATCGGTCAGTGGCTGTT |  |
| pmrK-RT-R | CCGCTTATCACGAAAGATCC |  |
| rpsL-RT-F | CCGTGGCGGTCGTGTTAAAGA |  |
| rpsL-RT-R | GCCGTACTTGGAGCGAGCCTG |  |

| **Isolates** | **MICs (µg/mL)^a^** | | | | | | | | | | | | |
| --- | --- | --- | --- | --- | --- | --- | --- | --- | --- | --- | --- | --- | --- |
|  | **PXB** | **TGC** | **MEM** | **IPM** | **CAZ** | **CRO** | **FEP** | **AMK** | **LVX** | **ATM** | **TZP** | **CSL** | **CZA** |
| CRKP-1 | 64 | 16 | 256 | 128 | >128 | >128 | >128 | >128 | >128 | >128 | >=128 | >=64 | **8/4** |
| CRKP-2 | 32 | **2** | 128 | 32 | >128 | >128 | >128 | >128 | 128 | >128 | >=128 | >=64 | **4/4** |
| CRKP-3 | 4 | **1** | 16 | 8 | >128 | 128 | >128 | **2** | **1** | 16 | >=128 | >=64 | >128/4 |
| CRKP-4 | 16 | **2** | 256 | 128 | >128 | >128 | >128 | >128 | 128 | >128 | >=128 | >=64 | **4/4** |
| CRKP-5 | 64 | **2** | 256 | 128 | >128 | >128 | >128 | >128 | 128 | >128 | >=128 | >=64 | **4/4** |
| CRKP-6 | 8 | **2** | 16 | 8 | >128 | >128 | >128 | >128 | 32 | >128 | >=128 | >=64 | **4/4** |
| CRKP-7 | 8 | **0.25** | 64 | 32 | 64 | 32 | >128 | **0.5** | 8 | 128 | >=128 | >=64 | **0.5/4** |
| CRKP-8 | 16 | 4 | 256 | 64 | >128 | >128 | >128 | >128 | 128 | >128 | >=128 | >=64 | **4/4** |
| CRKP-9 | 32 | **2** | 8 | 4 | 128 | >128 | >128 | >128 | >128 | >128 | >=128 | >=64 | **1/4** |
| CRKP-10 | 16 | **1** | 256 | 128 | >128 | >128 | >128 | >128 | >128 | >128 | >=128 | >=64 | >128/4 |
| CRKP-11 | 16 | **2** | 256 | 64 | >128 | >128 | >128 | >128 | 128 | >128 | >=128 | >=64 | **2/4** |
| CRKP-12 | 32 | 8 | 256 | 64 | >128 | >128 | >128 | >128 | 128 | >128 | >=128 | >=64 | **4/4** |
| CRKP-13 | 128 | **1** | 256 | 64 | >128 | >128 | >128 | 16 | 32 | >128 | >=128 | >=64 | **8/4** |
| CRKP-14 | 32 | **2** | 128 | 64 | >128 | >128 | >128 | >128 | 64 | >128 | >=128 | >=64 | **2/4** |
| CRKP-15 | 32 | **2** | 128 | 32 | 128 | >128 | >128 | >128 | 128 | >128 | >=128 | >=64 | **4/4** |
| CRKP-16 | 64 | **1** | 128 | 64 | >128 | >128 | >128 | >128 | 128 | >128 | >=128 | >=64 | **4/4** |
| CRKP-17 | 4 | **2** | 128 | 32 | >128 | >128 | >128 | >128 | 128 | >128 | >=128 | >=64 | **4/4** |
| CRKP-18 | 64 | **2** | 256 | 64 | >128 | >128 | >128 | >128 | 128 | >128 | >=128 | >=64 | **4/4** |
| CRKP-19 | 8 | **2** | 32 | 8 | 64 | 128 | >128 | **1** | 8 | 128 | >=128 | >=64 | **0.5/4** |
| CRKP-20 | 8 | **0.5** | 32 | 32 | 64 | 128 | >128 | **1** | 16 | 128 | >=128 | >=64 | **0.5/4** |
| CRKP-21 | 16 | **1** | 128 | 64 | 64 | >128 | >128 | >128 | 64 | >128 | >=128 | >=64 | **4/4** |
| CRECO-1 | 8 | **0.125** | 16 | 8 | >128 | >128 | >=32 | >128 | 64 | 64 | >=128 | >=64 | >128/4 |
| CRECO-2 | 8 | **0.125** | 64 | 16 | >128 | >128 | >=32 | **1** | 32 | >128 | >=128 | >=64 | >128/4 |
| CRECO-3 | 8 | **0.125** | 64 | 8 | >128 | >128 | 16 | **4** | 32 | **2** | 64 | >=64 | >128/4 |
| CRECO-4 | 4 | **0.25** | 32 | 8 | >128 | >128 | >=32 | >128 | 32 | >128 | >=128 | >=64 | >128/4 |
| CRECO-5 | 4 | **0.25** | 64 | 32 | >128 | >128 | >=32 | >128 | 32 | >128 | >=128 | >=64 | >128/4 |
| CRECO-6 | 4 | **1** | 16 | 8 | >128 | >128 | 16 | **2** | 8 | **1** | 64 | >=64 | >128/4 |
| CREC0-7 | 8 | **0.125** | 8 | 4 | >128 | 128 | 16 | **2** | 8 | 128 | >=128 | >=64 | >128/4 |
| CRECO-8 | 4 | **0.5** | 8 | 4 | >128 | >128 | >=32 | >128 | 64 | >128 | >=128 | >=64 | >128/4 |
| CRECO-9 | 8 | **0.25** | 8 | 16 | 64 | >128 | >=32 | **4** | 8 | **1** | >=128 | >=64 | >128/4 |

**Supplementary Table 4.** Minimum inhibitory concentrations (MICs) of polymyxin-resistant CRKP and CRECO isolates.

^a^PXB, polymyxin B; TGC, tigecycline; MEM, meropenem; IPM, imipenem; CAZ, ceftazidime; CRO, ceftriaxone; FEP, cefepime;

AMK, amikacin; LVX, levofloxacin; ATM, aztreonam; TZP, piperacillin-tazobactam; CSL, cefoperazone-sulbactam;

CZA, ceftazidime-avibactam. Numbers in boldface type indicate susceptibility according to CLSI/EUCAST breakpoints.

| Isolates | Chromosomal mutations^a^ | | | | |
| --- | --- | --- | --- | --- | --- |
|  | *mgrB* | *PhoP* | *PhoQ* | *PmrA* | *PmrB* |
| CRECO-1 | WT | Ile44Leu | Glu464Asp;  Ala482Thr | Ser29Gly | WT |
| CRECO-2 | WT | Ile44Leu; | WT | Ser29Gly;  Gly144Ser | Asp283Gly;  Tyr358Asn |
| CRECO-3 | WT | Ile44Leu; | Ser138Thr;  Ala482Thr | Ser29Gly; Asn219Arg;  Glu220Lys | His2Arg;  Ser138Asn;  Asp283Gly |
| CRECO-4 | WT | WT | Val386Leu | Ser29Gly;  Asn219Met | His2Arg |
| CRECO-5 | WT | WT | WT | Ser29Gly;  Asn219Met; | WT |
| CRECO-6 | WT | Ile44Leu; | WT | Ser29Gly;  Gly144Ser | Asp283Gly;  Tyr358Asn |
| CRECO-7 | WT | Ile44Leu; | Leu467Met | Ser29Gly;  Asn219Met; | His2Arg;  Asp283Gly |
| CRECO-8 | WT | Ile44Leu; | WT | Ser29Gly;  Gly144Ser | Asp283Gly；  Tyr358Asn |
| CRECO-9 | WT | WT | WT | Ser29Gly；  Asn219Ile；  Glu220Arg | WT |

**Supplementary Table 5.** Chromosomal mutations related to polymyxin resistance in CRECO isolates.

^a^Genetic determinants of resistance were detected by performing PCR with gene-specific primers. Mutations were identified by comparison with wild-type reference sequences [*E. coli* K12 MG1655 (accession: NC_000913.3) for *pmrAB*, *phoPQ*, *mgrB*]. The all mutations were predicted as neutral by PROVEAN. WT: wild-type.

| Genetic element | Size (bp) | G+C content (%) | Virulence genes | Resistance genes | Inc type | Conjugative modules |
| --- | --- | --- | --- | --- | --- | --- |
| Chromosome | 5493925bp | 57.32 | *fimA-K*, *mrkA-J，irp1-2*, *ybtA-X*, *entA-F*, *fepA-G*, *AcrAB*, *iroE*, *iroN*, *LPSrf*, *Capsule*, *T6SS*, *peg-344* | *bla*_SHV-182_, *fosA6* | NA | NA |
| pkp2007-VIR | 195008bp | 50.38 | *iucA-D*，*iutA*，*iroN*，*rmpA2*，*rmpA* | - | IncHI1B(pNDM-MAR)/repB | Partial |
| pkp2007-KPC | 127019bp | 52.75 | - | *bla*_CTX-M-65_, *rmtB*, *bla*_TEM-1B_, *bla*_SHV-12_, *bla*_KPC-2_ | IncFII（pHN7A8）/IncR | Partial |
| pkp2007-C | 87095bp | 53.95 | - | *qnrS1*, *bla*_LAP-2_, *tet(A)*, *catA2*, *sul2*, *dfrA14* | IncFII(pCRY) | **+** |
| pkp2007-D | 25542 bp | 56.82 | - | - | - | - |
| pkp2007-E | 11970 bp | 55.58 | - | - | ColRNAI_1 | - |
| pkp2007-F | 5596 bp | 51.13 | - | - | ColRNAI_1 | - |

**Supplementary Table 6.** Molecular characteristics of hypervirulent*K. pneumoniae* CRKP-5 and its plasmids.

**
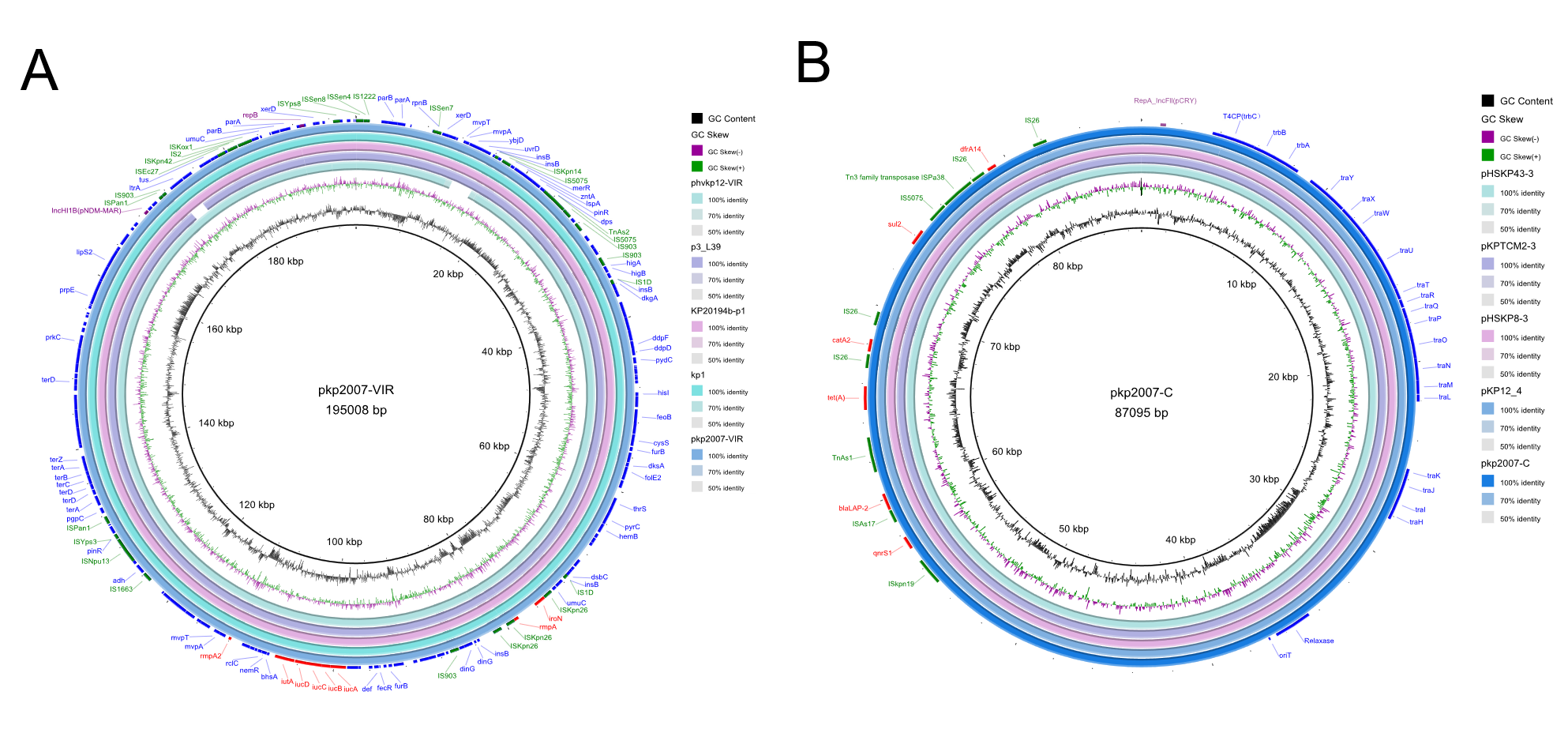
**

**Supplement Figure 1.** Comparative analysis of pkp2007-VIR and pkp2007-C with other similar plasmids in CRKP-5. Circular maps and genomic analysis of all insertion sequences distribution and antibiotic resistance genes. (A) BLASTn analysis showed that pkp2007-VIR backbone was similar to the classic virulence plasmid pLVPK,with over 99.94% nucleotide identity and 99% coverage ((e.g. CP103316.1, CP033955.1, CP054769.1 and CP146191.1). Virulence genes are indicated in red. Green arrows indicate insertion sequences. (B) BLASTn analysis showed that pkp2007-C was almost identical (100% query coverage, over 99.97% identity) to the plasmids pKPTCM2-3 (Zhejiang, China, CP118694.1), pKP12_4 (Zhejiang, China, CP082768.1), pHSKP43-3 (Shanghai, China, CP100101.1) and pHSKP8-3 (Shanghai, China, CP100090.1). ORFs encoding resistance genes are portrayed by red arrows. Green arrows indicate insertion sequences.

**Supplementary Table 7.** Molecular characteristics of polymyxin and tigecycline-resistant CRKP-1 and its plasmids.

| Features | Value for kp2020 | | | |
| --- | --- | --- | --- | --- |
|  | Chromosome | pkp2020 | pkp2020-B | pkp2020-C |
| Size | 5446350bp | 287004bp | 22008bp | 10361bp |
| G+C content (%) | 57.41 | 52.72 | 56.42 | 50.62 |
| Incapability group | / | IncFII(pHN7A8) /IncR/ IncFIB(K) | ColRNAI | ColRNAI |
| Resistance determinants | *bla*_SHV-182_，*fosA6* | *bla*_KPC-2_，*bla*_KPC-2_，*bla*_SHV-12_，*bla*_TEM-1B_，*rmtB*，*bla*_LAP-2_，*catA2*，*sul2*，*dfrA14*，*tet(A)*，*qnrS1*，*catA2* | / | / |
| Virulence factors | Type I fimbriae (*fimABCDEFGHIK*), Type 3 fimbriae(*mrkABCDFHIJ*), Yersiniabactin (*fyuA*, *irp1*, *irp2* and *ybtAEPQSTUX*), iutA, AcrAB (*acrAB*), ent siderophore (*entABCDEFS*, *fepABCDG*, *fes*), Capsule, Salmochelin(iroE, iroN), RcsAB(*rcsAB*), LPS rfb, T6SS-I (clpV/tssH, dotU/tssL, hcp/tssD, icmF/tssM, impA/tssA, ompA, sciN/tssJ, tli1, tssF-G, vasE/tssK, vgrG/tssI, and vipB/tssC), T6SS-II (clpV), T6SS-III(dotU, icmF, impA, impF, impG, impH, impJ, ompA, sciN, vgrG) | / | / | / |
| Conjugative modules | / | origin of transfer (oriT) region, relaxase gene, T4SS (traAEFLMX) | relaxase gene, coupling protein (T4CP) gene | origin of transfer (oriT) region, relaxase gene |

**Supplement Table 8.** The tigecycline resistance mechanism of the pan-resistant CRKP-1 strain.

| Strain | Mutations in tigecycline resistance related genes^a^ | | | | | | |
| --- | --- | --- | --- | --- | --- | --- | --- |
|  | TetA | ramR | AcrR | rpsJ | lon | *tmexCD*1*-toprJ*1 | TetX |
| CRKP-1 | I5R、V55M、I75V、T84A、S201A、F202S、V203F | **Ala17Thr** *194Lys | ISKpn26  insertion | WT | WT | Absent | Absent |

^a^Genetic determinants of resistance were detected by comparinng with wild-type reference sequences from *E.coli* plasmid RP1 (X00006) for *tet(A)* and *K. pneumoniae* MGH78578 (CP000647) for other genes.

The mutations predicted as novel and deleterious by PROVEAN were in bold.

WT: wildtype gene without mutations. Absent: the related genes were not present.

**Reference**

Borowiak, M., Baumann, B., Fischer, J., Thomas, K., Deneke, C., Hammerl, J. A., et al. (2020). Development of a Novel mcr-6 to mcr-9 Multiplex PCR and Assessment of mcr-1 to mcr-9 Occurrence in Colistin-Resistant Salmonella enterica Isolates From Environment, Feed, Animals and Food (2011–2018) in Germany. *Front. Microbiol.* 11, 80. doi: 10.3389/fmicb.2020.00080

Cannatelli, A., D’Andrea, M. M., Giani, T., Di Pilato, V., Arena, F., Ambretti, S., et al. (2013). In vivo emergence of colistin resistance in Klebsiella pneumoniae producing KPC-type carbapenemases mediated by insertional inactivation of the PhoQ/PhoP mgrB regulator. *Antimicrob. Agents Chemother.* 57, 5521–5526. doi: 10.1128/AAC.01480-13

Carattoli, A., Bertini, A., Villa, L., Falbo, V., Hopkins, K. L., and Threlfall, E. J. (2005). Identification of plasmids by PCR-based replicon typing. *J. Microbiol. Methods* 63, 219–228. doi: 10.1016/j.mimet.2005.03.018

Codjoe, F. S., Brown, C. A., Smith, T. J., Miller, K., and Donkor, E. S. (2019). Genetic relatedness in carbapenem-resistant isolates from clinical specimens in Ghana using ERIC-PCR technique. *PloS One* 14, e0222168. doi: 10.1371/journal.pone.0222168

Diancourt, L., Passet, V., Verhoef, J., Grimont, P. A. D., and Brisse, S. (2005). Multilocus sequence typing of Klebsiella pneumoniae nosocomial isolates. *J. Clin. Microbiol.* 43, 4178–4182. doi: 10.1128/JCM.43.8.4178-4182.2005

Jayol, A., Poirel, L., Brink, A., Villegas, M.-V., Yilmaz, M., and Nordmann, P. (2014). Resistance to colistin associated with a single amino acid change in protein PmrB among Klebsiella pneumoniae isolates of worldwide origin. *Antimicrob. Agents Chemother.* 58, 4762–4766. doi: 10.1128/AAC.00084-14

Johnson, T. J., Bielak, E. M., Fortini, D., Hansen, L. H., Hasman, H., Debroy, C., et al. (2012). Expansion of the IncX plasmid family for improved identification and typing of novel plasmids in drug-resistant *Enterobacteriaceae*. *Plasmid* 68, 43–50. doi: 10.1016/j.plasmid.2012.03.001

Pishnian, Z., Haeili, M., and Feizi, A. (2019). Prevalence and molecular determinants of colistin resistance among commensal Enterobacteriaceae isolated from poultry in northwest of Iran. *Gut Pathog.* 11, 2. doi: 10.1186/s13099-019-0282-0

Poirel, L., Walsh, T. R., Cuvillier, V., and Nordmann, P. (2011). Multiplex PCR for detection of acquired carbapenemase genes. *Diagn. Microbiol. Infect. Dis.* 70, 119–123. doi: 10.1016/j.diagmicrobio.2010.12.002

Portal, E. A. R., Sands, K., Farley, C., Boostrom, I., Jones, E., Barrell, M., et al. (2024). Characterisation of colistin resistance in Gram-negative microbiota of pregnant women and neonates in Nigeria. *Nat. Commun.* 15, 2302. doi: 10.1038/s41467-024-45673-6

Quan, J., Li, X., Chen, Y., Jiang, Y., Zhou, Z., Zhang, H., et al. (2017). Prevalence of mcr-1 in Escherichia coli and Klebsiella pneumoniae recovered from bloodstream infections in China: a multicentre longitudinal study. *Lancet Infect. Dis.* 17, 400–410. doi: 10.1016/S1473-3099(16)30528-X

Rebelo, A. R., Bortolaia, V., Kjeldgaard, J. S., Pedersen, S. K., Leekitcharoenphon, P., Hansen, I. M., et al. (2018). Multiplex PCR for detection of plasmid-mediated colistin resistance determinants, mcr-1, mcr-2, mcr-3, mcr-4 and mcr-5 for surveillance purposes. *Eurosurveillance* 23, 17–00672. doi: 10.2807/1560-7917.ES.2018.23.6.17-00672

Russo, T. A., Olson, R., Fang, C.-T., Stoesser, N., Miller, M., MacDonald, U., et al. (2018). Identification of Biomarkers for Differentiation of Hypervirulent Klebsiella pneumoniae from Classical K. pneumoniae. *J. Clin. Microbiol.* 56, e00776-18. doi: 10.1128/JCM.00776-18

Tartof, S. Y., Solberg, O. D., Manges, A. R., and Riley, L. W. (2005). Analysis of a uropathogenic Escherichia coli clonal group by multilocus sequence typing. *J. Clin. Microbiol.* 43, 5860–5864. doi: 10.1128/JCM.43.12.5860-5864.2005

Wang, X., Liu, Y., Qi, X., Wang, R., Jin, L., Zhao, M., et al. (2017). Molecular epidemiology of colistin-resistant Enterobacteriaceae in inpatient and avian isolates from China: high prevalence of *mcr*-negative *Klebsiella pneumoniae*. *Int. J. Antimicrob. Agents* 50, 536–541. doi: 10.1016/j.ijantimicag.2017.05.009
